# Human infection studies: Key considerations for challenge agent development and production

**DOI:** 10.12688/wellcomeopenres.17869.1

**Published:** 2022-04-22

**Authors:** Shobana Balasingam, Sarah Meillon, Cecilia Chui, Alex Mann, Carine La, Charlotte L. Weller, Deborah F. King, Emma Smith

**Affiliations:** 1 Infectious Diseases | Prevention, Wellcome Trust, UK, London, N1 2BE, UK; 2 Clinical Sciences, hVIVO, London, E1 2AX, UK; 3 Global Health Innovative Technology Fund, Tokyo 106-0032, Japan; 4 HIC-Vac, Imperial College London, National Heart & Lung Institute (NHLI), London, W2 1PG, UK

**Keywords:** human challenge study, human infection study, challenge agents, GMP, challenge agent production

## Abstract

Human infection (or challenge) studies involve the intentional administration of a pathogen (challenge agent) to volunteers. The selection, isolation, development and production of the challenge agent is one of the first steps in developing a challenge study and critical for minimising the risk to volunteers. Regulatory oversight for this production differs globally. Manufacturing agents within a Good Manufacturing Practice (GMP) facility reduces the risk of the manufacturing process by including processes such as confirming the identity of the challenge agent and ascertaining that it’s pure and free from impurities. However, in some cases it’s not possible or feasible to manufacture to GMP standards, for example where the challenge agent requires an intermediate vector for growth. There is lack of clear guidance on what the minimum requirements for high-quality safe manufacture outside of GMP facilities should be and here we describe the development of a considerations document for the selection and production of challenge agents to meet this need.

## Introduction

Human infection studies, also known as human challenge studies, are where volunteers are intentionally given a carefully considered dose of a pathogen (known as the challenge agent). These innovative models can be used to study host-pathogen interactions and disease progression, test the efficacy and down-select vaccines and drugs in development, or be used as proof of concept (POC) studies for testing novel medications ^1, 2^. One of the advantages of these studies is that they require only a small number of healthy adult volunteers to gain confidence on efficacy before progressing to larger more costly Phase III field trials ^3^.

Historically, human infection studies have been few in number and largely been conducted in Europe and America. However, since the turn of the century the number of human infection studies conducted has risen dramatically ^4^, facilitated in part by significant technological advances that have increased the scientific value of this research. In the last decade there has also been an increase in their geographical spread, with *Plasmodium falciparum*, and more recently *Plasmodium vivax* ^5^ and *Pneumococcus* ^6^ challenge studies being conducted in Africa and Asia.

In recent years, human challenge study data has contributed to the licensure of influenza antivirals and a live oral cholera vaccine approved by the U.S. Food and Drug Administration (FDA) ^7^. Human challenge data also contributed to the World Health Organisation (WHO) prequalification of Bharat Biotech’s Typbar TCV®, a typhoid conjugate vaccine, which facilitated its procurement and distribution in UNICEF, Pan-American Health Organization (PAHO) and GAVI supported countries ^8, 9^. The recent severe acute respiratory syndrome coronavirus 2 (SARS-CoV-2) human challenge study performed in the UK has launched discussions on the use of these studies as part of future pandemic preparedness plans and it is imperative therefore that frameworks and guidelines exist to ensure these studies are conducted as safely as can be ^10^.

The selection, isolation, development and production of the challenge agent is the first step towards establishing a challenge study and the regulatory oversight for this production differs globally. In the US the challenge agent is considered a biologic and is subject to regulation under federal law (section 351 of the Public Health Service Act and Food, Drug and Cosmetic Act). The challenge agent must therefore (i) be manufactured under Good Manufacturing Practice (GMP) conditions where possible, (ii) satisfy the FDA regulations of safety, purity and potency, and (iii) have detailed information on the provenance and manufacture as part of the required Investigational New Drug application (IND). Other countries and regions have similar regulatory requirements, for example the European Union (EU). However, in other parts of the world challenge agents are not viewed as medicines or biologics and are thus not regulated in the same way a medicinal product is. For example, the UK’s Medicines and Healthcare products Regulatory Agency (MHRA) do not regulate the manufacture of challenge agents, leaving the responsibility for ensuring the challenge agent is produced stringently and safely to the Sponsor ^11, 12^. In countries without legal regulation of challenge agent manufacturing, they are often manufactured under the principles of GMP but without adhering to all the specific requirements. However, there is an absence of clear guidance of what the minimum requirements should be.

A meeting organized by International Alliance For Biological Standardization (IABS) in 2019 ^11^ focusing on the quality requirements for challenge agents explored how the FDA accepts challenge agents that have not been manufactured under full GMP conditions on a case-by-case basis. An example of where this might be applied is when the challenge agent requires an intermediate vector for growth e.g. *Plasmodium falciparum* with the use of mosquitos ( *Anopheles*) and *Schistosoma mansoni* with the use of snails ( *Biomphalaria glabrata).* In these circumstances researchers are strongly encouraged to engage with the FDA but, similar to countries without legal regulation, might benefit from clear guidance of what the minimum requirements should be.

The increase in human challenge studies being established in low-income or middle-income countries (LMIC) and the desirability for challenge agents to reflect naturally occurring and epidemiologically relevant pathogen strains means there is an increased likelihood that they will be manufactured in conditions where full GMP is not possible. Currently the challenge studies being conducted in African countries use challenge agents that have been manufactured in the US or Europe and have already been administered to volunteers in “characterisation" studies in these regions confirming their safety and utility. In fact, recently published guidance on human challenge studies by the Pharmacy and Poisons Board in Kenya state that this is a prerequisite before the challenge study can be established in Kenya ^13^.

It is useful to note that challenge studies have a good safety profile and serious adverse events (SAEs) are rare, as observed in a review of influenza challenge studies that identified only one SAE after inoculation of 2462 volunteers ^14^. However rare, an SAE has the potential to harm both volunteers and confidence in whole field (as demonstrated by the aforementioned SAE, which halted influenza challenge studies in the US for nearly a decade ^14, 15^), so it is critical to minimise the risks of an SAE occurring. 

Whilst manufacturing challenge agents within a GMP facility does not necessarily mean the challenge agent is safe from a pathogenicity perspective, the procedures are designed to minimise the risk of an SAE caused by the manufacturing process. GMP processes assist with confirming the identity of the challenge agent, ascertaining that the agent is pure and free from impurities (e.g. contaminating unwanted pathogens), and characterising the amount and reproducibility of the product, where possible ^8^. Developing clear guidance setting out the minimum requirements for challenge agents manufactured outside of GMP would help ensure they mitigate risks posed to volunteers by the manufacturing process to the same degree. 

As funders of human challenge studies globally, Wellcome and HIC-Vac proactively decided to fund the development of a considerations document for the selection and production of challenge agents with relevance to challenge agents manufactured within a GMP facility as well as when not. This document, titled ‘Considerations on the Principles of Development and Manufacturing Qualities of Challenge Agents For Use In Human Infection Models’, was prepared by hVIVO in consultation with a consortium of global experts in the human challenge field including those from Africa, Asia, USA, EU and global regulators ^16^. It outlines considerations for the development, characterisation and manufacture of infectious challenge agents, promoting volunteer safety whilst maximizing access to challenge agents and challenge models in LMICs and academic institutions globally. The document presents basic principles for the selection, characterisation, manufacture, quality control and storage of challenge agents for international reference. It is hoped that these considerations will be used across high-, middle- and low-income countries for the safe production of challenge agents by trained personnel with appropriate facilities, quality control measures and other best practices.

To make the considerations more user friendly for human challenge researchers, a workshop was also held in December 2021 to produce a Smart Practices Document which covers the practical and logistical considerations with reference to the Consideration Document prepared. This will be made available online for comment by the research community to improve practices for producing challenge agents safe for use in challenge studies protecting both the volunteer and the field.

## Data availability

No data are associated with this article.
